# Effect of Microbial Consortium Constructed with Lignolytic Ascomycetes Fungi on Degradation of Rice Stubble

**DOI:** 10.3390/jof9050567

**Published:** 2023-05-13

**Authors:** Kallinkal Sobha Sruthy, Livleen Shukla, Aditi Kundu, Sandeep Kumar Singh, Hissah Abdulrahman Alodaini, Ashraf Atef Hatamleh, Gustavo Santoyo, Ajay Kumar

**Affiliations:** 1 Division of Microbiology, ICAR-Indian Agricultural Research Institute, New Delhi 110012, India; 2 Division of Agricultural Chemicals, ICAR-Indian Agricultural Research Institute, New Delhi 110012, India; 3 Department of Botany and Microbiology, College of Science, King Saud University, P.O. Box 2455, Riyadh 11451, Saudi Arabia; 4 Instituto de Investigaciones Químico-Biológicas, Universidad Michoacana de San Nicolás de Hidalgo, Morelia 58030, Mexico; 5 Centre of Advanced Study in Botany, Banaras Hindu University, Varanasi 221005, India

**Keywords:** rice stubble, *Aspergillus*, lignolytic enzymes, fungi, biodegradation

## Abstract

Microbial degradation is an effective, eco-friendly and sustainable approach for management of the rice residue. After harvesting a rice crop, removal of stubble from the ground is a challenging task, that forces the farmers to burn the residue in-situ. Therefore, accelerated degradation using an eco-friendly alternative is a necessity. White rot fungi are the most explored group of microbes for accelerated degradation of lignin but they are very slow in growth. The present investigation focuses on degradation of rice stubble using a fungal consortium constructed with highly sporulating ascomycetes fungi, namely, *Aspergillus terreus*, *Aspergillus fumigatus* and *Alternaria* spp. All three species were successful at colonizing the rice stubble. Periodical HPLC analysis of rice stubble alkali extracts revealed that incubation with ligninolytic consortium released various lignin degradation products such as vanillin, vanillic acid, coniferyl alcohol, syringic acid and ferulic acid. The efficiency of the consortium was further studied at different dosages on paddy straw. Maximum lignin degradation was observed when the consortium was applied at 15% volume by weight of rice stubble. Maximum activity of different lignolytic enzymes such as lignin peroxidase, laccase and total phenols was also found with the same treatment. FTIR analysis also supported the observed results. Hence, the presently developed consortium for degrading rice stubble was found to be effective in both laboratory and field conditions. The developed consortium or its oxidative enzymes can be used alone or combined with other commercial cellulolytic consortia to manage the accumulating rice stubble effectively.

## 1. Introduction

Recently, microbial-based degradation of lignocellulosic materials has been gaining momentum due to their being eco-friendly, non-toxic and economical. In previous studies, numerous fungal strains have been reported for effective biodegradation of lignocellulosic materials by their secretory products or enzymes [1].

Rice stubble burning is the most obvious option for many farmers, particularly in areas where the subsequent crop must be sown within a short duration. However, burning releases large quantities of particulates and greenhouse gases, including methane, carbon dioxide, nitrous oxide and CO, which are known to have deleterious effects on human health. On average, particulate matter emission in India due to the open burning of rice straw is 144, 719 Mt per year [2]. However, open burning of straw affects the air quality, by releasing gases such as CO_2_ and CO to the environment [3,4]. 

Rice stubble, a lignocellulosic material, is mainly constructed with cellulose, lignin and hemicellulose. Lignin contributes to its recalcitrance by binding the cellulose strands and hemicellulose together. Out of these three biopolymers, lignin is the most resistant to degradation due to its structural complexity [5]. Despite its highly complex structure, numerous microorganisms possess the inherent ability to decompose lignin [6]. 

Since a large part of the organic carbon in the stubble is shielded by lignin, research on biodegradation is imperative. In the last few years, various fungal groups such as brown rot fungi (*Gloeophyllum trabeum* and *Coniophora puteana*) and white rot fungi (*Ganoderma* spp., *Lentinula edodes*, *Ceriporiopsis subvermispora, Phlebia radiata* or *Pleurotus* spp.) have been reported for the efficient degradation of lignin [7,8,9]. In addition, strains such as *Trametes villosa* have also been reported for the presence of genes encoding lignin-modifying enzymes [10]. Similarly, the strain *Irpex lacteus* is also considered as a promising source of lignocellulolytic enzymes [11]. 

Deconstruction of lignin begins with the oxidation of lignin side chains that contain the methoxy group to molecules such as CO_2_ and hydroxyl groups. Later, depolymerization of lignin results in the release of individual phenolic groups and subsequent metabolism of these phenolic groups occurs inside microbial cells [12]. This oxidative degradation of lignin involves several enzyme activities. Among the enzymes, extracellular enzymes such as lignin peroxidases (LiPs), laccase and manganese peroxidases (MnPs) are well studied. The role of LiPs and MnPs in lignin decomposition is the best understood. However, the exact role of other enzymes is yet to be revealed [13].

So far, several studies have been conducted on the collective management of rice residues [14,15,16]. However, some of these methods may leave a recalcitrant stubble portion in the field undecomposed. Moreover, most of the lignin-degrading fungi being investigated are white rot fungi or fungi belonging to Agaricales, which are slow growing and leave recalcitrant rice stubble and roots undecomposed under field conditions. Keeping this in mind, the present study focuses on the degradation of rice stubble, particularly emphasizing lignin deconstruction by the comparatively fast-growing ascomycetes fungi.

## 2. Materials and Methods

**Figure 1:**
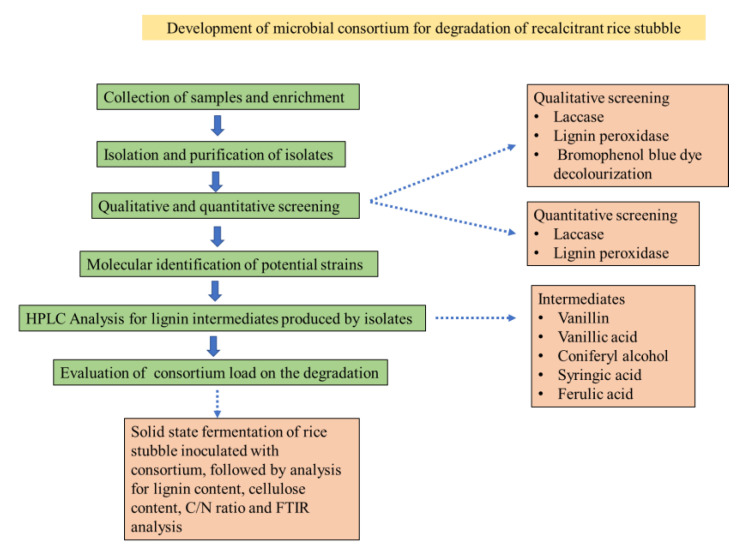
Research methodology used in this study.

The research methodology used in this study is summarized in Figure 1.

### 2.1. Isolation and Screening of Lignin-Degrading Fungi

Degrading rice stubble and soil were collected, chopped and kept in RMM medium [17], supplemented with 1% guaiacol, tannin or 0.1% lignin in different flasks and incubated at 30 °C for 14 days. After enrichment for 14 days, cultures were isolated using serial dilution and spread plate method on RMM medium supplemented with 1% guaiacol, tannin and 0.1% lignin, respectively. Fungal isolates showing distinct morphology were selected, purified and maintained on potato dextrose agar slants. Isolates obtained were screened qualitatively for laccase activity [18], lignin peroxidase activity (modified method of Egger) [19] and bromophenol blue plate assay [20]. The selected fungal isolates were inoculated on 2% malt extract agar plates for 7 days at 30 °C. After seven days, a well was made in the center of the plates and bottom was sealed with molten agar. For detection of laccase production, 1 mL freshly prepared guaiacol solution (2- methoxyphenol; 1% *v/v* was prepared by the mixing of 1 mL guaiacol in 95% ethanol) was added to the well and the plates were kept in the dark. The plates were observed after 10 h. Development of a red to purple color indicated laccase production. For detection of lignin peroxidase enzyme, 0.5 mL pyrogallol aqueous solution (1.0% *w*/*v*) was added to the wells and plates were kept at room temperature in the dark for ten hours. Development of golden yellow to brown color indicated the presence of lignin peroxidase activity. Presence of clear zones in the media containing bromophenol blue indicated the ability of the isolates to degrade bromophenol blue dye. Shortlisted isolates were subjected for the quantitative estimation of lignocellulolytic enzymes. For this, the isolates were grown in sterilized Reese mineral medium (50 mL) supplemented with 1% (*w*/*v*) rice stubble chopped in 2 cm lengths. Flasks were incubated for 14 days at 30 °C under submerged fermentation conditions and the broth was filtered using Whatman filter paper No. 41. The filtrate was used to estimate lignin peroxidase [21] and laccase [22]. For determination of lignin peroxidase activity, the enzyme filtrate (0.5 mL) was added to cuvettes containing 0.5 mL 0.05 mol. L^−1^ citrate buffer with pH 4.8. To this, 50 µL 0.05 mol. L^−1^ Azure-B and 50 µL of 10 millimol. L^−1^ H₂O₂ were added. H₂O₂ was not added to blank. Absorbance was recorded for 180 s at 651 nm after every 30 s interval. However, laccase activity was determined by measuring the change in absorbance at 436 nm using 5 millimol. L^−1^ ABTS as substrate. For this, enzyme filtrate (1 mL) was taken in a cuvette, along with 1 mL of 0.05 millimol. L^−1^ citrate buffer (pH 4.8). For initiating the assay, 0.2 mL of 5 millimol. L^−1^ ABTS was added to the cuvette containing sample. ABTS was not added to the blank. The absorbance was measured for 180 s at 436 nm at intervals of 30 s. 

Three cultures that produced a good quantity of lignolytic enzymes were selected for further study. First, the morphology of the isolates was studied by staining the cultures with lactophenol cotton blue and observing them under an optical microscope under magnifications 400× and 1000×. The fungal biomass in terms of N-acetyl glucosamine was quantified using method given by Aidoo et al. [23]. Then, DNA was isolated using the C-TAB method to identify the species. In brief, 4 g of fungal mycelium was taken, working C-TAB buffer was added, and it was incubated at 60 °C for 1 h in the water bath and then subjected to centrifugation for 10 min at 1000 rpm. Further, 500 µL of supernatant was collected into a new centrifuge tube and an equal volume of chloroform: isoamyl alcohol (24:1) was added. Then, the tubes were centrifuged at 10,000 rpm for 2 min. Finally, the supernatant was transferred into a fresh centrifuge tube, adding 300 µL isopropanol (0.6 vol) and 50 µL sodium acetate (0.1 vol).

The tubes were incubated overnight at −20 °C. The next day, tubes were centrifuged at 10,000 rpm for 10 min at 4 °C. The supernatant was thrown carefully without disturbing the pellet in the bottom of the tube. Further, tubes were washed by adding 500 ul of 75% ethanol and centrifuged at 10,000 rpm for 10 min at 40 °C. Alcohol was discarded carefully and the pellets were air-dried. TE buffer was added to them, and they were kept overnight to dissolve the DNA at 40 °C and stored at −20 °C). The concentration of DNA was checked in Nano-Drop and used for PCR amplification. Primers ITS-1(5′-TCCGTAGGTGAACCTGCGG-3′) and ITS-4 (5′-TCCTCCGCTTATTGATATGC-3′) were used for amplification [24]. PCR was undertaken with optimized conditions: 95 °C for 5 min (initial denaturation), 30 cycles of 95 °C for 30 s, 50 °C for 30 s and 72 °C for 60 s, followed by final elongation at 72 °C for 10 min. The equivalent amount of MilliQ water was used as a negative control. PCR products were visualized by observing the horizontal agarose gel (1.2%) amended with 0.002% ethidium bromide, following the standard protocols. The gel images were obtained using Gel Documentation System (AlphaImager 1220, Alpha Innotech Corporation, Sengliandria, CA, USA) equipped with a CCD camera. Sequencing of ITS rRNA was outsourced from the Eze Diagnon sequencing facility, Coimbatore, India, using the same primer set. 

To confirm the ability of fungal species to colonize recalcitrant rice stubble, 5 g recalcitrant rice stubble was taken in 100 mL of RMM medium and sterilized in an autoclave. The promising isolates were inoculated separately on rice stubble (rice variety PB 1121) and incubated at 30 ± 2 °C in a BOD incubator; the growth of fungal agents was observed after 7 days. Then the rice stubble was taken out and a representative sample was stained to observe under the microscope. A cross-section of root stubble was prepared and stained with lactophenol cotton blue to visualize the growth of isolates on it.

### 2.2. Analysis for Intermediates of Lignin Degradation

Rice stubble was chopped into 2 cm lengths and inoculated with the fungal consortium by adding two plugs of fully grown fungal cultures and 70% moisture was maintained. However, the negative control was maintained without culture inoculation. The rice stubble was kept for decomposition under solid-state fermentation conditions at room temperature. Lignin was extracted on 0, 7, 14, 21 and 28th days using 0.1 mol. L^−1^ NaOH. This filtrate was used for the HPLC analysis using the HPLC system (Alliance, Waters 2998 Corp., Milford, NZ, USA) equipped with e 2695 separation module, quaternary pump and a 2998 Photo Diode Array detector with an “empower 2” software program. However, during HPLC analysis, 20 µL of sample and C18 column (SUPELCO^®^, USA) of (25 cm × 4.6 mm, 5 µm I.D) stationary phase were used to separate the extractives. Acetonitrile: H_2_O with 0.1% formic acid was used as a solvent in the gradient condition and the peaks were visualized at a wavelength of 264 nm. 

For the analysis, reference standards such as coniferyl alcohol, vanillin, vanillic acid, syringic acid and ferulic acid were purchased from Merk^®^ India Ltd. (Mumbai, India) and Sigma^®^ chemicals (Bangalore, India). The concentrations of intermediates were calculated from the areas of peaks appearing at respective retention times by referring to the calibration curve.

### 2.3. Evaluation of Consortium Load on the Degradation

Three fungal isolates were grown in the potato dextrose broth to study the effect of the consortium. The cultures were churned and viable count was adjusted to 10^10^ cfu/mL and mixed in a 1:1:1 ratio. Further, Pusa decomposer, a commercial waste decomposing fungal consortium, was procured from the Division of Microbiology, ICAR-IARI, New Delhi. Pusa decomposer is a fungal consortium used for degradation of crop residues [25]. In this study, this consortium was used as a positive control. For the experiment, 500 g of rice stubble of PB 1121 was packed in a polybag and inoculum applied at various concentrations. Small pores were made in the polybag to facilitate aeration. Inoculated rice stubble was incubated for 45 days. 

For estimation of total organic carbon, a silica crucible was weighed and rice stubble (a known amount) was taken and ignited at 600 °C using muffle furnace for four hours [26]. Remaining ash was weighed and the amount of organic carbon was calculated as

Percentage of Carbon = (100 − % ash)/1.74

Nitrogen content in the sample was determined by Micro-Kjeldahl method [27]. Sample (100 mg) was weighed and put in a digestion flask. Concentrated sulfuric acid (5 mL) and catalyst mixture (100 mg) was added to the flask and digestion process was performed until the solution became colorless. The contents from the digestion flask were transferred and the volume made up to 25 mL. Distillation was performed in distillation unit with 5 mL digested sample and 10 mL 40% NaOH solution. Further, ammonia released was trapped in 25 mL 1% boric acid solution containing mixed indicator. Once the ammonia was trapped, this was back titrated with 0.01 mol. L^−1^ hydrochloric acid solution. A blank was maintained only with distilled water.

Estimation of total phenols was performed using the method described previously [28]. Next, cellulose content of the dried ground sample was estimated by the procedure given by [29]. Finally, lignin content was determined following the NREL (National Renewable Energy Laboratory, Golden, CO, USA) protocol [30]. However, the Fourier transform infrared (FTIR) analysis was used to examine the compositional changes through functional groups of compounds in rice straw after degradation. 

For the analysis, initially samples were dried and ground into powder form and mixed with oven-dried KBr powder to prepare a pellet by pressing the mixture with a pressure of 15,000 psi. A Perkin Elmer Spectrum BX FTIR measured the entire reflectance spectrum. At a resolution of 4 cm^−1^, 32 scans per spectra were accumulated, with wave numbers ranging from 4000 to 400 cm^−1^. During the experiment, three biological replicates of each sample were analyzed. However, background correction was produced by employing a KBr pellet as a sample background with identical instrument settings. In addition, peak height values were calculated by measuring the spectra’s transmittance. 

### 2.4. Statistics

The experiments were carried out in a completely randomized design (CRD) and all the samples were processed with three biological replicates. Further, the results were analyzed using one-way ANOVA depending on the means to evaluate the significances or differences with software SPSS.16 version. The value *p* < 0.05 was considered significant using Tukey’s test. 

## 3. Results and Discussion

### 3.1. Isolation, Screening and Identification of Potential Ligninolytic Fungi

Decaying rice residue is a rich source for the growth of fungi having ligninolytic potential [31]. Laccase and lignin peroxidase are common enzymes involved in lignin degradation [32]. Laccase is a multi-copper enzyme that uses molecular oxygen as an electron acceptor. It oxidizes phenolic compounds in lignin and other aromatic compounds such as aromatic amines and benzenothiols [33,34,35]. Lignin peroxidase is an enzyme that oxidizes phenolic aromatic substrates and several non-phenolic lignin model compounds non-specifically in the presence of hydrogen peroxide [36]. 

Enrichment of the collected rice stubble in lignin, tannin and guaiacol for 14 days selectively supported the growth of 19 fungal isolates. Among the 19, only 10 isolates were able to produce laccase and 14 isolates produced lignin peroxidase. Ten isolates decolorized bromophenol blue and only three isolates degraded tannic acid. It was observed that most fungal isolates showed lignin peroxidase activity compared to the laccase enzyme. Eight isolates with more than two positive results in qualitative analysis were selected for further screening quantitatively and the three isolates among them exhibited the highest production of lignin peroxidase and laccase (Table 1). 

The highest laccase activity was observed in isolate LN-14 (0.203 IU/mL) and the highest lignin peroxidase activity was shown by LN-1 (0.265 IU/mL). Isolate LN-19 was also recorded with a promising quantity of these two enzymes. 

The DNA sequences of the isolates LN-1 showed homology with *Aspergillus terreus* (Accession number-MT165896.1). Kumari et al. [37] also reported lignin peroxidase production from *Aspergillus terreus*. Similarly, LN-14 showed homology with *Aspergillus fumigatus* (Accession number-MT165898.1). Jin and Ning [38] also studied laccase production from a different strain, *Aspergillus fumigatus* AF1. LN-19 shows a maximum of 100% homology with *Alternaria alternata* (Accession number-MT165897.1). 

### 3.2. Colonization of Selected Lignolytic Fungal Isolates on Recalcitrant Rice Stubble

**Figure 2:**
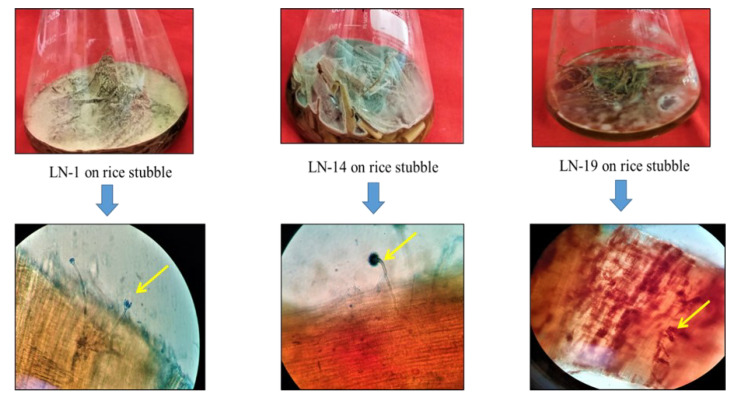
Successful colonization of the isolates on rice stubble (**upper**) and microscopic view of colonization at 400× (**lower**).

The isolates successfully colonized rice stubble, which was validated by microscopic observation under 400× magnification (Figure 2). The literature reports suggest that *A. terreus* and *A. fumigatus* could utilize rice straw as substrates [39,40]. In the present study, *A. terreus* (LN-1) and *A. fumigatus* (LN-14) were successfully established on rice stubble within 72 h after inoculation. At the same time, growth of *A. alternata* was comparatively slow and fully colonized on the rice stubble only after five days. Under 400× microscopic visualization, growth of *A. terreus* on rice stubble as a substratum with a compact conidial head was clearly visible. The green echinulate conidia of *A. fumigatus* were visible on rice stubble during the growth stage. The microscopic view provided a clear image of the morphology of the growing fungal hyphae on rice-straw-bearing uniseriate conidial heads.

On the other hand, *A. alternata* was also successful at colonization on rice stubble and produced brown-colored large conidia during its growth. Additionally, high estimated fungal biomass in terms of N-acetyl glucosamine 35.68, 36.31 and 28.00 mg g^−1^ for *A. terreus*, *A. fumigatus* and *Alternaria alternata*, respectively, confirmed the ability of the isolates to colonize on rice stubble. Glucosamine content in the material is an indirect measure of biomass content, thus indicating the extent of colonization of the particular organism [41].

### 3.3. Study on Efficiency of the Developed Fungal Consortium on Rice Stubble Degradation

HPLC analysis is an efficient and time-saving method for regular analysis of metabolites of lignin degradation [42]. Grasses typically contain 15–25% lignin and the estimated quantity of lignin in rice straw is 12% [43,44]. Lignin degradation performed by the fungal consortium was periodically analyzed by estimating intermediates formed after solid state fermentation of rice straw with the consortium. The consortium of three lignolytic fungal isolates was found to be effective for degrading rice stubble lignin, which was confirmed through the HPLC analysis of alkali extracts from degraded rice stubble. Lignin degradation and conversion of lignin monomers are tracked by various authors using LC-MS and HPLC [45,46]. The results showed presence of various metabolites produced after degradation. Intermediates of lignocellulose degradation were present in the treatments, where rice stubble was inoculated with the consortium. However, a negligible quantity (traces) of intermediates was detected in the negative control. Lignin is constructed with phenyl propane units linked together by ether bonds or carbon–carbon linkages. The main constituents of lignin include coniferyl, coumaryl and sinapyl alcohols [5]. In fact, during chemical modification, a wide variety of low molecular weight compounds, vanillic acid, vanillin, coniferyl aldehyde and syringic acid, were produced [21]. 

**Figure 3:**
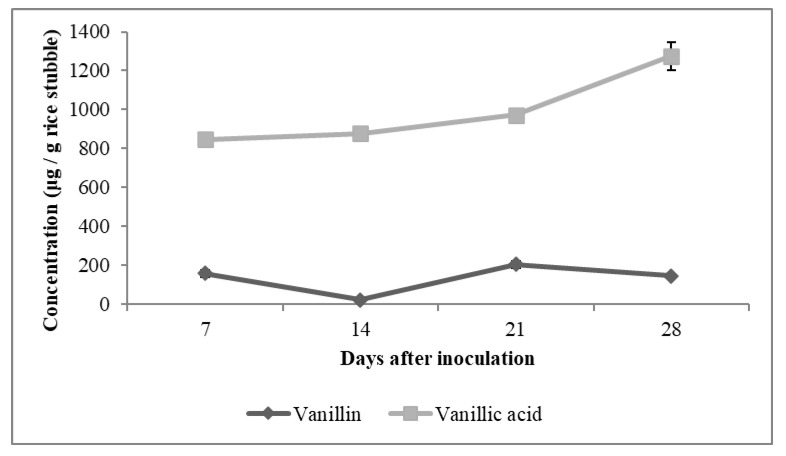
Vanillin and vanillic acid profile during growth of fungal consortium (*A. terreus*, *A. fumigatus*, *Alternaria alternata*) on rice stubble.

**Figure 4:**
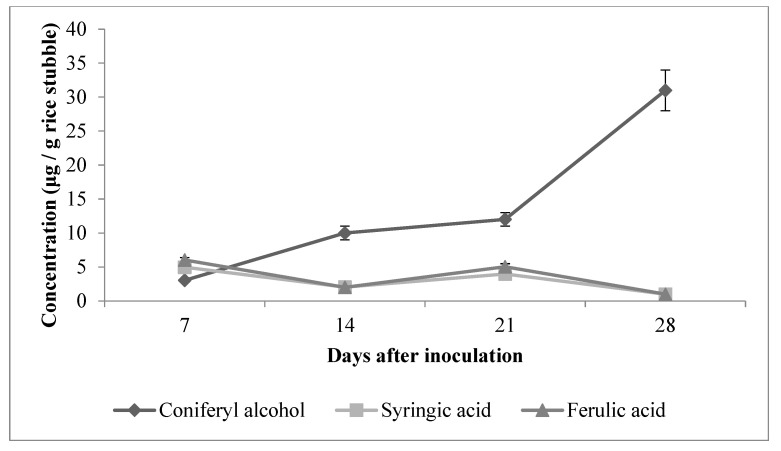
Profile of other degradation products during growth of fungal consortium (*A. terreus*, *A. fumigatus*, *Alternaria alternata*) on rice stubble.

In the present study, the highest quantity of vanillin (205 µg g^−1^ rice stubble) was detected on the 21st day of incubation. Intermediates such as vanillic acid and coniferyl alcohol showed a steady increase from the 7th day to the 28th day after inoculation (Figure 3 and Figure 4). 

**Figure 5:**
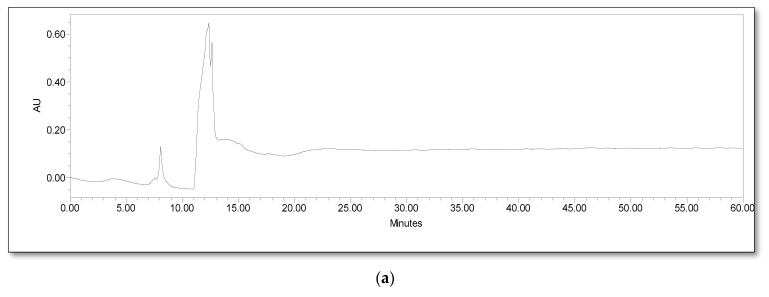

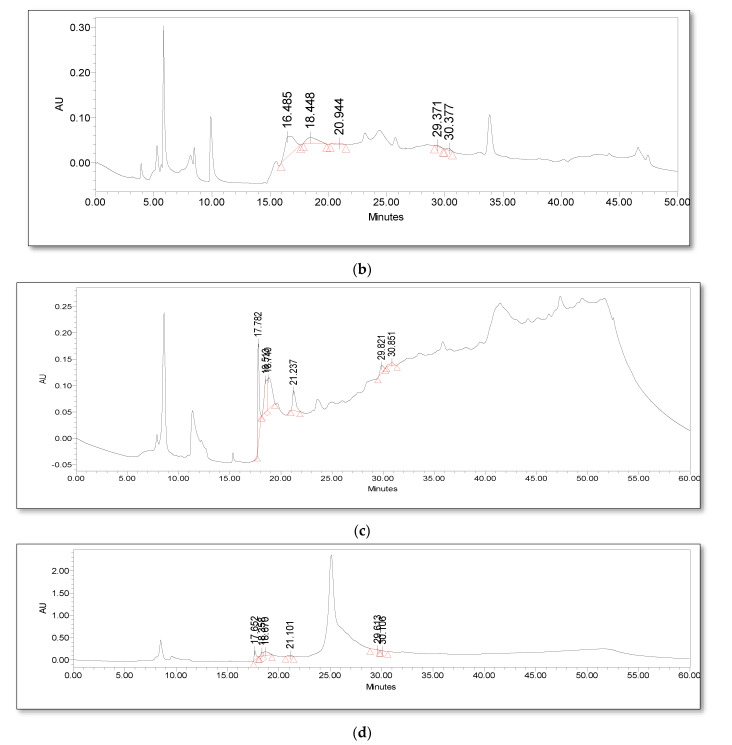
HPLC chromatogram illustrating time course analyses of rice stubble inoculated with lignolytic fungal consortium. Figure (**a**) is the HPLC chromatogram of alkali extracts from day 0 samples, showing no peaks representing lignin degradation products. Figure (**b**–**d**) is the HPLC chromatogram of alkali extracts from day 14, 21 and 28 showing numerous peaks of products from lignin degradation.

As vanillic acid is a monocyclic aromatic compound derived from lignin by an ortho-cleavage of the protocatechuic acid branch through the β-ketoadipate pathway, it is the most reported degradation metabolite of lignin [47]. Other common degradation products of lignin are 4-hydroxy-benzoic acid, 4-hydroxy-3-methoxy-benzoic acid (vanillic acid), 4-hydroxy-3-methoxy-benzaldehyde (vanillin), 4-hydroxy-3,5-dimethoxy-benzoic acid (syringic acid), 4-hydroxy-3-methoxy-cinnamyl alcohol (coniferyl alcohol) and ferulic acid [42,48]. It was also observed from the HPLC analysis data that, as the degradation progressed, concentration of vanillic acid also increased. The maximum concentration of vanillic acid was estimated as 1275 µg g^−1^ of stubble on the 28th day of incubation. The highest concentrations of ferulic acid (6 µg g^−1^) and syringic acid (5 µg g^−1^) were found on the 7th day of incubation. The concentration of these two phenolic acids was found to be decreased slightly on the 14th day and again subsequently increased on the 21st day of fungal inoculation. Apart from this, the HPLC chromatogram showed many unidentified peaks emerging as the fungi grew on the rice stubble during the period under observation and assay (Figure 5).

### 3.4. Effect of Newly Developed Lignolytic Fungal Inoculum on Rice Stubble Degradation

It is known that the concentration of inoculum influences the rate of decomposition considerably. This is mainly because the amount of inoculum can influence the time microorganisms require to colonize the substrate. Generally, spores are used as inoculum and large inoculum size will shorten the time required for colonization on substrates [49,50]. In the present study, rice stubble degradation was performed by keeping the treatment without inoculum as control and different concentrations of inoculum (5%, 10% and 15% *v*/*w*) were applied. Degradation parameters were studied up to 45 days.

Initially, the rice stubble contained 11.56% lignin and 42.87% cellulose. The carbon to nitrogen (C:N) ratio of rice stubble before degradation was 83.19. During degradation, carbon content tends to decrease due to degradation of high molecular weight carbohydrates, whereas nitrogen content tends to increase due to accumulation of nitrate. This will lead to a narrow C:N ratio of material after degradation [51]. Table 2 shows the biochemical parameters of rice stubble after 45 days of inoculation with different concentrations of newly developed lignolytic consortium. The lignin content was found to be highest in treatment without inoculum, indicating significantly less degradation of lignin. This reduction in lignin content in the uninoculated treatment is due to the native microflora of rice stubble. There was a significant difference in the lignin content of rice stubble inoculated with 10% and 15% newly developed lignolytic consortium. The highest reduction in lignin was recorded in rice stubble inoculated with 15% lignolytic consortium (lignin content 5.31%) followed by 10% consortium (lignin content 6.63%). A minimum reduction in lignin was observed in control (lignin content 9.77%), where no fungal inoculation was carried out. However, the highest reduction in cellulose was observed in treatment inoculated with Pusa decomposer (17.36%), followed by rice stubble inoculated with 15% newly developed lignolytic inoculum. Thus, the already existing consortium, Pusa decomposer, effectively degraded cellulose, not lignin. Reduction in cellulose content was minimal in the control, where no fungal culture was inoculated, which was maintained with native microflora. The minimum reduction in cellulose and lignin in the control can be attributed to the native microflora of rice stubble. C/N ratio of rice stubble was found to be 43.46 in treatment inoculated with 15% newly developed lignolytic inoculum, which was significantly different from the control where no culture was inoculated.

As lignin is polyphenolic in nature, aromatic lignin intermediates are released during the degradation of lignocellulose. In wheat straw extract, free phenolics are detected [52]. Total phenols were found to be highest in rice stubble when inoculated with 15% newly developed lignolytic consortium (571 µg g^−1^), followed by 10% of the same consortium (563 µg g^−1^). The phenol content of all the treatments was significantly higher than the control, where no fungal culture was inoculated in which phenol content was recorded as 397 µg g^−1^ of rice stubble (Table 3). Similarly, Yesilada et al. [53] also reported that the dosage of inoculum significantly affects biodegradation and removal of phenols from waste water. Fungal biomass colonized on rice straw was estimated in terms of N-acetyl glucosamine. The highest fungal biomass was found to be in treatment inoculated with Pusa decomposer, an existing fungal consortium (92.88 mg g^−1^), followed by a newly developed lignolytic consortium applied at 15% (*w*/*v*) concentration (89.26 mg g^−1^).

Previous studies stated that *A. fumigatus* show high laccase production in pre-treated rice straw [39]. Laccase activity was not detected in treatment without any inoculum and was highest in the treatment with 15% newly developed lignolytic consortium (0.95 IU g^−1^). Similar trends were observed with lignin peroxidase; treatment with 15% newly developed lignolytic consortium recorded the highest lignin peroxidase activity (1.54 IU g^−1^). In solid-state fermentation of switch grass, maximum lignin degradation of 52% was obtained when 5 mL of inoculum was applied [54]. The present study was also supported by the results reported by Li et al. [54] which described the effect of substrate inoculum ratio (ranging from 2 to 6) on degradation. They reported that anaerobic digestion quickly initiated at a substrate inoculum ratio of 6. Maximum production of methane was observed when a substrate inoculum ratio was 2 [54]. In the present study, the degradation efficiency was found to increase with the concentration of inoculum.

#### FTIR Analyses of Rice Stubble Inoculated Lignolytic Consortium

**Figure 6:**
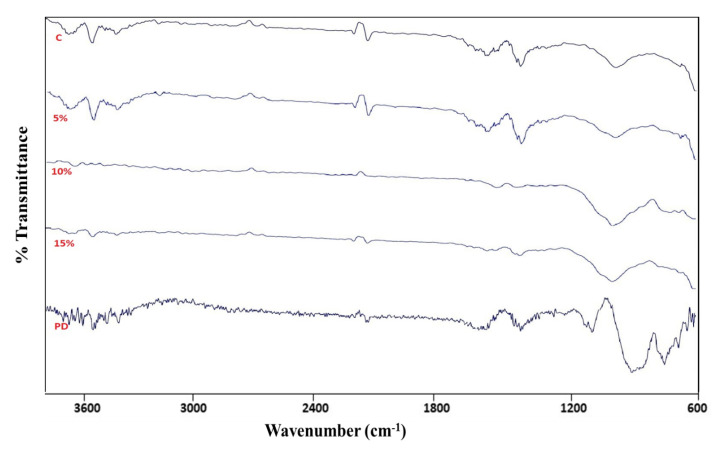
FTIR of rice stubble treated with different concentrations of lignolytic consortium.

FTIR analysis of rice stubble treated with different concentrations of newly developed lignolytic inoculum showed a significant plunge in the intensities indicating the change in lignin and related functional groups (Figure 6). A sharp peak at 1225 cm^−1^ was attributed to the -CO stretching of guaiacol and syringyl ring. Another peak at 1441 cm^−1^ in different treatments was assigned to C-H vibrations, originating due to aliphatic -CH_2_ or phenolic -OH groups. Further, stretching at 3364 cm^−1^ in the treatments was assigned to phenolic -OH. Treatments showed characteristic carboxylic acidic (-COO) and carbonyl (-CO) functional groups at 2952 cm^−1^ and 1685 cm^−1^ in the FTIR spectra corresponded to phenolic acids and benzaldehydes, respectively. As the degradation of lignin proceeds, methoxy groups disappear in a steady manner [55]. A slight reduction in transmittance in this region indicates degradation of -OH and -CH_3_ moieties. Rice stubble treated with a 15% lignolytic consortium showed more significant reduction in transmittance in this specified region. Similar observation on reduction in transmittance due to faster degradation of -OH and CH_3_ has also been often reported in the literature [56,57,58]. Rice stubble treated with 15% lignolytic consortium showed a similar pattern of transmittance % with the specified functional groups. A visible difference in wave number was observed between corresponding bands, originating due to the stretching and bending of selected functional groups of treated samples with different concentrations of ligninolytic fungal consortium and control.

## 4. Conclusions

This study explored different soil ascomycetes for degradation of rice stubble. Various members of genera *Aspergillus* and *Alternaria* are well known phytopathogens and affect the plant health adversely. However, some of the strains of these genera are beneficial with regard to degradation of crop waste residue. In the present investigation, fungi belonging to the genera *Aspergillus* and *Alternaria* were found to be efficient in managing this accumulated residue. The three fungi used were successful at colonizing rice stubble and efficient at the degradation of rice stubble. The presence of different lignin degradation products such vanillin and vanillic acid proved the lignin degradation by the newly developed consortium. As the dosage of inoculum increased, degradation of lignin and cellulose was also observed to be increased. So, the present investigation showed that the newly isolated ascomycetes can effectively degrade rice stubble. In future, these fungal cultures or their purified lignolytic enzymes can also combine with other compatible and efficient microorganisms to manage various crop residues. These fungi can also be used for production of value-added lignin intermediates from rice stubble.

## Figures and Tables

**Table 1:** Laccase and lignin peroxidase activity of the potential strains isolated.

Isolates	Laccase Activity (IU/mL)	LiP Activity (IU/mL)
LN-1	0.176 ± 0.0020 ^b^	0.265 ± 0.0014 ^a^
LN-7	0.056 ± 0.0016 ^e^	0.034 ± 0.0028 ^g^
LN-9	0.045 ± 0.0012 ^f^	0.067 ± 0.0008 ^d^
LN-13	0.067 ± 0.0021 ^d^	0.045 ± 0.0017 ^f^
LN-14	0.203 ± 0.0043 ^a^	0.192 ± 0.0020 ^c^
LN-15	ND	0.051 ± 0.0004 ^e^
LN-17	0.036 ± 0.0016 ^g^	0.031 ± 0.0012 ^g^
LN-19	0.154 ± 0.0017 ^c^	0.217 ± 0.0057 ^b^

Note: Equal letters in the same column indicate that there is no significant difference between the means (*p* > 0.05). ND, Not Detected.

**Table 2:** Lignin, cellulose and C/N ratio of rice stubble after 45 days of decomposition.

Treatments	Lignin (%)	Cellulose (%)	C/N
Control (rice stubble)	9.77 ± 0.233 ^a^	39.48 ± 1.115 ^a^	57.93 ± 0.738 ^a^
Rice stubble + 5% LC	8.74 ± 0.176 ^b^	32.03 ± 0.808 ^b^	50.71 ± 1.406 ^b^
Rice stubble + 10% LC	6.63 ± 0.015 ^d^	20.96 ± 1.005 ^c^	43.80 ± 0.810 ^c^
Rice stubble + 15% LC	5.31 ± 0. 214 ^e^	19.87 ± 0.905 ^cd^	43.46 ± 0.765 ^c^
Rice stubble + PD	7.50 ± 0.090 ^c^	17.36 ± 0.568 ^d^	45.21 ± 0.796 ^bc^

Note: LC, Lignolytic Consortium; PD, Pusa Decomposer. Equal letters in the same column indicate that there is no significant difference between the means (*p* > 0.05).

**Table 3:** Biochemical parameters of rice stubble after 45 days of decomposition.

Treatments	Total Phenols (µg g^−1^)	Fungal Biomass (mg g^−1^ Substrate)	Laccase Activity (IUg^−1^)	LiP Activity (IU g^−1^)
T1—rice stubble	397 ± 9.165 ^e^	37.6 ± 1.331 ^e^	ND	ND
T2—rice stubble + 5% MC	472 ± 4.000 ^d^	59.61 ± 1.075 ^d^	0.28 ± 0.010 ^c^	0.45 ± 0.010 ^d^
T3—rice stubble + 10% MC	563 ± 4.041 ^b^	81.56 ± 1.246 ^c^	0.91 ± 0.026 ^b^	1.37 ± 0.025 ^b^
T4—rice stubble + 15% MC	571 ± 4.041 ^a^	89.26 ± 1.170 ^b^	0.95 ± 0.015 ^a^	1.54 ± 0.010 ^a^
T5—rice stubble + PD	483 ± 4.041 ^c^	92.88 ± 1.023 ^a^	0.24 ± 0.015 ^e^	0.98 ± 0.015 ^c^

Note: MC, Microbial Consortium; PD, Pusa Decomposer. The alphabetical letters in the superscript indicate the significant difference between the treatments, with “a” being significantly highest at *p* = 0.05. ND, Not Detected.

## Data Availability

Not Applicable.
